# Features of trastuzumab-related cardiac dysfunction: deformation analysis outside left ventricular global longitudinal strain

**DOI:** 10.3389/fcvm.2024.1291180

**Published:** 2024-01-19

**Authors:** Giang M. Nhat, Nguyen H. Hai, Vo T. Duc, Ho H. Q. Tri, Chau N. Hoa

**Affiliations:** ^1^ Department of Cardiac Intensive Care and Cardiomyopathy, Nhan dan Gia Dinh Hospital, Ho Chi Minh City, Vietnam; ^2^ University of Medicine and Pharmacy at Ho Chi Minh City, Ho Chi Minh City, Vietnam; ^3^ Diagnostic Imaging Department, University Medical Center of Ho Chi Minh City, Ho Chi Minh City, Vietnam; ^4^ Heart Institute, Ho Chi Minh City, Vietnam; ^5^ Faculty of Medicine, University of Medicine and Pharmacy at Ho Chi Minh City, Ho Chi Minh City, Vietnam

**Keywords:** early cancer therapy-related cardiotoxicity, trastuzumab, left ventricular global longitudinal strain, left ventricular ejection fraction, left atrial longitudinal strain, right ventricular longitudinal strain

## Abstract

**Background:**

Cancer therapy-related cardiac dysfunction due to trastuzumab has been well-known for many years, and echocardiographic surveillance is recommended every 3 months in patients undergoing trastuzumab treatment, irrespective of the baseline cardiotoxicity risk. However, the potential harm and cost of overscreening in low- and moderate-risk patients have become great concerns.

**Objectives:**

This study aimed to identify the incidence of early cancer therapy-related cardiac dysfunction (CTRCD) and the behaviours of left and right heart deformations during trastuzumab chemotherapy in low- and moderate-risk patients.

**Methods:**

We prospectively enrolled 110 anthracycline-naïve women with breast cancer and cardiovascular risk factors who were scheduled to receive trastuzumab. The left ventricular ejection fraction (LVEF), left ventricular global longitudinal strain (LV-GLS), and right ventricular and left atrial longitudinal strains were evaluated using echocardiography at baseline, before every subsequent cycle and 3 weeks after the final dose of trastuzumab. The baseline risk of CTRCD was graded according to the risk score proposed by the Heart Failure Association (HFA) Cardio-Oncology Working Group and the International Cardio-Oncology Society (ICOS). CTRCD and its severity were defined according to the current European Society of Cardiology (ESC) guidelines.

**Results:**

Twelve (10.9%) patients had asymptomatic CTRCD. All CTRCD occurred sporadically during the first 9 months of the active trastuzumab regimen in both low- and moderate-risk patients. While CTRCD was graded as moderate severity in 41.7% of patients and heart failure therapy was initiated promptly, no irreversible cardiotoxicity or trastuzumab interruption was recorded at the end of follow-up. Among the left and right heart deformation indices, only LV-GLS decreased significantly in the CTRCD group during the trastuzumab regimen.

**Conclusions:**

CTRCD is prevalent in patients with non-high-risk breast cancer undergoing trastuzumab chemotherapy. Low- and moderate-risk patients show distinct responses to trastuzumab. The LV-GLS is the only deformation index sensitive to early trastuzumab-related cardiac dysfunction.

## Introduction

Although the introduction of HER-2 inhibitors, including trastuzumab, pertuzumab and lapatinib in the chemotherapy regimen of HER-2 positive invasive breast cancer, has dramatically improved the remission and mortality rate in both early and metastatic settings, cancer-related cardiac dysfunction (CTRCD) due to this targeted therapy has been a concern, especially in combined anthracycline and trastuzumab therapy (1). The subsequent protocol of trastuzumab following anthracycline can lower the risk of cardiotoxicity compared to the conjunctive regimen; however, CTRCD remains prevalent from 3% to 19% (2). When anthracycline is safely removed from the chemotherapy protocol for HER2-positive breast cancer in recent clinical trials, the risk of CTRCD has been reduced from 0.4% to 3.2% (3, 4). Although anthracycline is no longer preferred for most trastuzumab-containing regimens, echocardiography surveillance every 3 months is recommended to detect subclinical or asymptomatic CTRCD during chemotherapy for all patients according to the current European Society of Cardiology (ESC) guidelines, irrespective of baseline cardiotoxicity risk (5). This recommendation is suggested according to the echocardiography protocol of trastuzumab clinical trials with limited validation in low and high-baseline risk categories (6). Since echocardiography surveillance is performed in a manner of one-size-fits-all, the potential harmfulness and cost regarding overscreening in low- and moderate-risk patients has become a great concern. Identifying the incidence of CTRCD in low- and moderate-risk patients receiving trastuzumab without anthracycline may indicate the need for guideline-directed echocardiography in these non-high-risk groups.

Compared to serial LVEF monitoring, frequent assessment of left ventricular global longitudinal strain (LV-GLS) is recommended to detect CTRCD earlier during trastuzumab chemotherapy and promptly initiate medical treatment for heart failure; however, the role of LV-GLS in this clinical scenario is mainly extrapolated from anthracycline-based treatment data (5). The incidence of asymptomatic CTRCD defined by LV-GLS reduction has not been identified in most contemporary pivotal trials of trastuzumab regimen (7). Furthermore, the involvement of the right ventricular and left atrial longitudinal strain in CTRCD has recently become an active area of research. Although some studies have shown that right ventricular global longitudinal strain (RV-GLS) (8), right ventricular free-wall longitudinal strain (RV-FWLS) (9) or left atrial reservoir strain (LASr) (10) decreased significantly with the trastuzumab regimen, most patients were pre-exposed to anthracycline. In contrast to anthracycline, primary myocyte injury does not occur and dose-dependent cardiotoxicity has not been clearly elucidated in patients receiving trastuzumab monotherapy. Neuregulin ERBB pathway, which was found to be connected to the stress response of the heart, is proposed to play a principal role in this type II chemotherapy-related cardiac dysfunction (11). While the main features of trastuzumab-induced cardiotoxicity include the lack ultrastructural damage and reversibility (2), it remains unclear whether the right ventricle and left atrium are affected concurrently with the left ventricle and which cardiac chamber is more sensitive to trastuzumab-induced cardiotoxicity. Therefore, this study aimed to identify the incidence of early CTRCD and the behaviours of left and right heart deformations during trastuzumab chemotherapy in low- and moderate-risk patients by incorporating all echocardiographic deformation indices measured at baseline and before every subsequent trastuzumab cycle in an anthracycline-naive breast cancer cohort with cardiovascular risk factors.

## Methods

### Design

This multicentre prospective cohort study enrolled 110 consecutive female patients who received trastuzumab as adjuvant or neoadjuvant chemotherapy for newly diagnosed breast cancer (stages I–IV) at the Nhan Dan Gia Dinh Hospital and Oncology Hospital, Ho Chi Minh City, Vietnam between 01 September 2020 and 31 December 2022. All patients received an 18-cycle trastuzumab regimen with a 21-day inter-cycle interval. Patients were eligible for inclusion in this study if they presented at least one of the following cardiovascular risk factors: ≥60 years of age, hypertension, diabetes mellitus, dyslipidaemia, atrial fibrillation, obesity (BMI > 30 kg/m^2^), or chronic kidney disease. The exclusion criteria included severe valvular heart disease and poor image quality on echocardiogram, defined as ≥2 inadequate visualised myocardial segments (in an 18-segment model) or previous exposure to anthracycline. Patients were evaluated for standard demographic and clinical data, HFA-ICOS risk scores for CTRCD (6), echocardiography findings, and medical therapy at baseline. All patients underwent clinical examination and standard echocardiography before every trastuzumab cycle and 3 weeks after the final dose, irrespective of their HFA-ICOS risk categories. The primary end point was CTRCD occurrence during trastuzumab therapy. The severity of CTRCD was stratified according to the recent ESC guidelines, in which mild CTRCD was defined as a new relative decline in GLS by >15% from the baseline value with LVEF ≥50%, and moderate CTRCD as a new LVEF reduction by ≥10 percentage points to an LVEF 40%–49% with or without a new relative decline in GLS by >15% from the baseline value (5). When LVEF reduction was confirmed by two consecutive echocardiography to <50%, moderate and severe CTRCD was recorded. If CTRCD was detected, trastuzumab was interrupted only in the severe presentation, whereas heart failure medications with ACE-i/ARB, beta-blockers and MRA were initiated promptly in moderate and severe settings. Regarding CTRCD reversibility after trastuzumab treatment, LVEF recovery was categorised as follows: fully reversible (to within 5 percentage points of the baseline) or partially reversible (improved by ≥10 percentage points but remained >5 percentage points below baseline) (12). This study was conducted in accordance with the principles of the Declaration of Helsinki and was approved by the ethical committee of the University of Medicine and Pharmacy at Ho Chi Minh City (approval number: NCT04547465), and all patients provided informed consent. Patients or the public were not involved in the design, or conduct, or reporting, or dissemination plans of our research.

### Two-dimensional echocardiography

Echocardiography was performed at a resting condition using a Philips Affiniti ultrasound system (Philips Healthcare, Andover, MA, USA) by a single examiner, according to the current guidelines of the American Society of Echocardiography and the European Association of Cardiovascular Imaging (12, 13). Data from four-, three-, and two-chamber and right ventricular-focused apical views acquired in three consecutive cardiac cycles at a frame rate of >50 fps were stored in raw DICOM format for offline analysis. Images were obtained at baseline (before the first trastuzumab cycle), before every subsequent cycle, and 3 weeks after the completion of chemotherapy. Nineteen echocardiographic examinations were performed on each patient during the study period. All images were transferred to a core laboratory and computed by two cardiologists (H. H. N. and D. T. V.) who were blinded to the patients' clinical data. Right ventricular strains (RV-FWLS and RV-GLS) and R-R gating left atrial strains (LASr, LAScd and LAScd) were assessed using right ventricular-focused and apical four-chamber views, respectively. Strain analyses by speckle-tracking (LV-GLS, RV-FWLS, RV-GLS, LASr, LAScd and LAScd) and semiautomatic LVEF calculations were performed using a Philips aCMQ (QLAB 15.0, Philips Healthcare, Andover, MA, USA). The LA border was automatically defined in the LASr, LAScd, and LAScd procedures, and zero strain reference was set at left ventricular end-diastole. LASr, LAScd and LASct were measured as the difference of the strain value at mitral valve opening minus ventricular end-diastole, the difference of the strain value at the onset of atrial contraction minus mitral valve opening, and the difference of the strain value at ventricular end-diastole minus onset of atrial contraction, respectively (13). The strain indices at each trastuzumab cycle were compared with the corresponding values at baseline, and the difference was defined as the relative change [relative change at each cycle = (current strain value–baseline strain value)/baseline strain value]. The baseline strain index was identical to the strain value before cycle 1.

### Statistical analysis

Data were provided as means ± standard deviation when normally distributed, medians and interquartile ranges for skewed distributions and frequencies and percentages for categorical variables. A Chi-squared or unpaired *t-*test was used to compare the demographic data, cardiovascular risk factors, HFA-ICOS risk levels and medical therapy between the mild CTRCD, moderate CTRCD and no-CTRCD groups. A paired *t-*test was used to demonstrate significant changes in ΔLV-GLS, ΔRV-FWLS, ΔRV-GLS, ΔLASr, ΔLAScd and ΔLAScd between independent groups at each anthracycline cycle. Statistical analyses were performed using IBM SPSS Statistics version 27 (IBM Corp., Armonk, NY, USA) and R version 4.0.3 (R Foundation for Statistical Computing, Vienna, Austria). Two-sided *P*-values were used, and statistical significance was set at *P *< 0.05.

## Results

### Patient characteristics and CTRCD incidence

**Figure 1:**
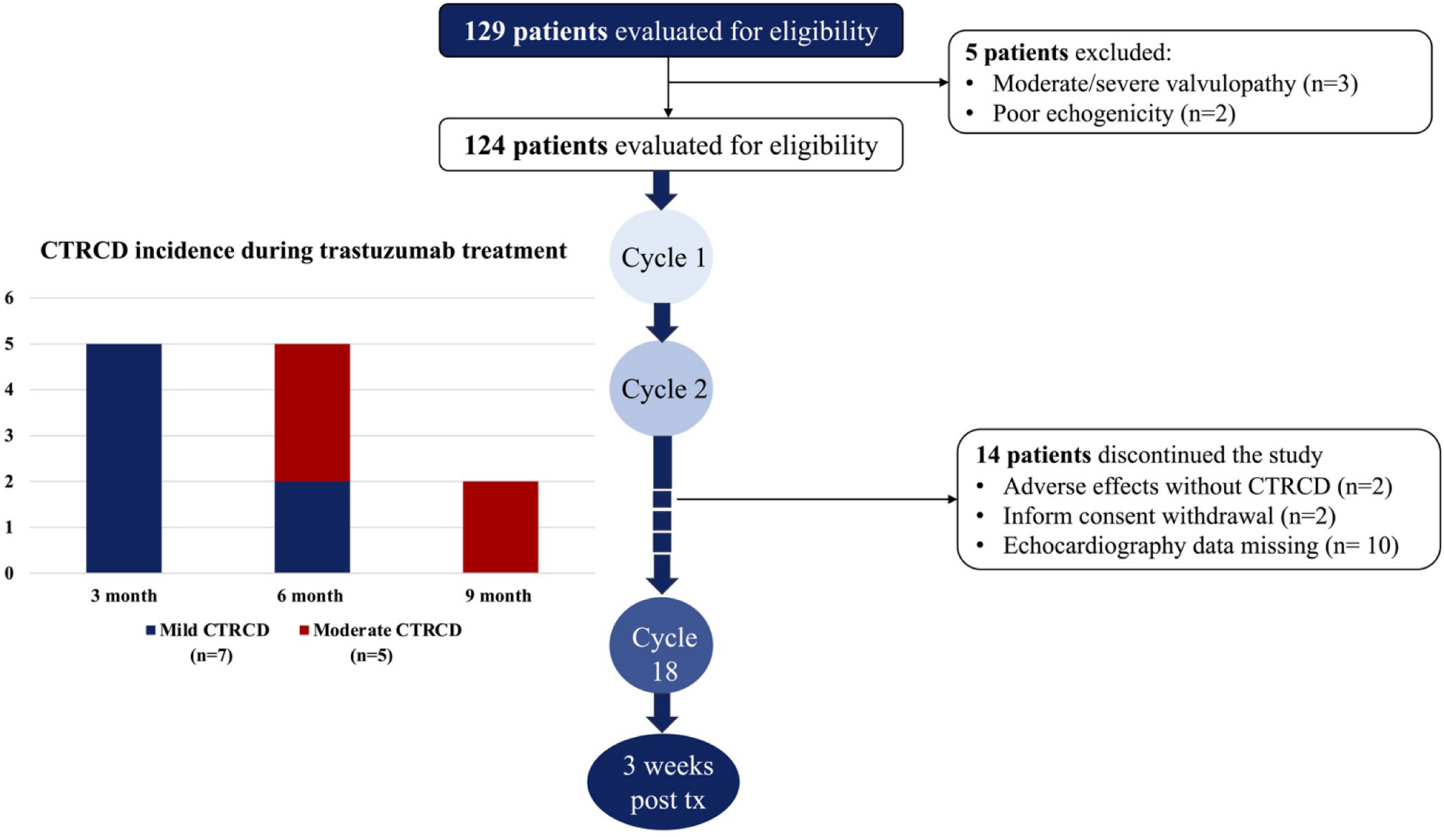
Study flow chart and incidence of CTRCD during trastuzumab chemotherapy. All CTRCD occured during the first 9 months of the active trastuzumab regimen. In the first three months, only mild CTRCD was recorded while moderate CTRCD developed in the second and third­three months.

Of the 110 patients with breast cancer and cardiovascular risk factors, 12 (10.9%) developed CTRCD during trastuzumab chemotherapy and were asymptomatic at the time of detection. All CTRCD events were defined as mild or moderate severity with LVEF ≥40%, and no CTRCD event was recorded after 9 months of trastuzumab chemotherapy. Moderate CTRCD developed later than mild CTRCD, only after the first 3 months (Figure 1).

At baseline, patients' average age was 60.2 ± 8.3 years (33–78 years). No patient presented with LVEF <50%, prior CTRCD, heart failure, cardiomyopathy or a history of myocardial infarction with or without coronary revascularization. Hypertension was the predominant cardiovascular risk factor in 69 (62.7%) patients, followed by dyslipidaemia in 43 (39.1%), diabetes mellitus in 25 (22.7%), chronic kidney disease in 10 (9.1%) and obesity in five (4.5%). All cardiovascular risk factors at baseline were similar between the CTRCD and non-CTRCD groups. Given that all the patients had cardiovascular risk factors, 28 (25.4%) received cardioprotective therapy with ACE-i/ARB (23.9%) and beta-blockers (11.8%) before trastuzumab treatment. Baseline left ventricular function (LVEF and LV-GLS), right ventricular strain (RV-GLS and RV-FWLS) and left atrial mechanics (LASr, LAScd, and LASct) assessed using echocardiography did not differ between the no-CTRCD and CTRCD groups. Moderate CTRCD did not have a lower LV-GLS value or other strain parameters at baseline than mild CTRCD or no CTRCD. The baseline characteristics of the patients in the CTRCD and non-CTRCD groups are summarised in Table 1.

**Table 1:** Patient characteristics.

	Total (*n* = 110)	No-CTRCD (*n* = 98)	CTRCD (*n* = 12)			*P*_1_	*P*_2_	*P*_3_
			All CTRCD (*n* = 12)	Mild CTRCD (*n* = 7)	Moderate CTRCD (*n* = 5)			
Age	60.2 (8.3)	60.5 (7.9)	57.9 (11.4)	54.4 (10.8)	62.8 (11.4)	0.316	0.268	0.677
BMI	24.2 (2.8)	24.2 (2.9)	24.1 (2.3)	24.7 (2)	23.2 (2.6)	0.894	0.231	0.435
Breast cancer stage								
Stage 0–1	9 (8.2)	7 (7.1)	2 (16.7)	1 (14.3)	1 (20)	0.327	0.531	0.704
Stage 2	57 (51.8)	50 (51)	7 (58.3)	4 (57.1)	3 (60)			
Stage 3	35 (31.8)	32 (32.7)	2 (25)	2 (28.6)	1 (20)			
Stage 4	9 (8.2)	9 (9.2)	0 (0)	0 (0)	0 (0)			
Cardiovascular risk factor								
Hypertension	69 (62.7)	62 (63.3)	7 (58.3)	4 (57.1)	3 (60)	0.738	0.921	0.883
Diabetes mellitus	25 (22.7)	21 (21.4)	4 (33.3)	2 (28.6)	2 (40)	0.543	0.679	0.331
Atrial fibrillation	1 (0.9)	1 (1)	0 (0)	0 (0)	0 (0)	0.725	NA	0.82
Dyslipidemia	43 (39.1)	39 (39.8)	4 (33.3)	2 (28.6)	2 (40)	0.665	0,679	0.943
Obesity (BMI > 30 kg/m^2^)	5 (4.5)	4 (4.1)	1 (8.3)	1 (10)	0 (0)	0,213	0,377	0.665
Chronic kidney disease	10 (9.1)	10 (10.2)	0 (0)	0 (0)	0 (0)	0.246	N.A	0.452
HFA-ICOS category								
Low risk	58 (52.7)	51 (52)	7 (58.3)	7 (100)	0 (0)	0.678	**0.001**	**0.023**
Moderate risk	52 (47.3)	47 (48)	5 (41.7)	0 (0)	5 (100)			
Baseline medical therapy								
ACE-i	12 (10.9)	12 (12.2)	0 (0)	0 (0)	4 (80)	0.199	**0.004**	0.162
ARB	16 (14.5)	12 (12.2)	4 (33.3)	0 (0)	1 (20)	0.05	0.217	0.489
Beta blocker	13 (11.8)	12 (12.2)	1 (8.3)	1 (14.3)	0 (0)	0.692	0.377	0.344
Baseline echocardiography								
LVEF (%)	63.6 (5.3)	63.8 (5.31)	61.5 (5.4)	61.1 (5.5)	62 (5.7)	0.175	0.801	0.517
LV-GLS (%)	−18.9 (1.3)	−18.9 (1.3)	−19.3 (1.4)	−19.3 (1.7)	−19.1 (1.2)	0.385	0.790	0.653
>18%	83 (75.5)	73 (74.5)	10 (83.3)	6 (85.7)	4 (80)	0.456	0.743	0.567
16%–18%	26 (23.6)	24 (24.5)	2 (16.7)	1 (14.3)	1 (20)	0.982	0.878	0.978
<16%	1 (0.9)	1 (1)	0 (0)	0 (0)	0 (0)	N.A	N.A	N.A
RV-FWLS (%)	−28.3 (2)	−28.3 (2)	−28.8 (2.3)	−28.4 (1.1)	−29.3 (3.4)	0.487	0.617	0.557
RV-GLS (%)	−25.1 (19.1)	−25.4 (20.2)	−23.4 (2.2)	−23.5 (2)	−23.3 (2.6)	0.362	0.868	0.378
LASr (%)	44.3 (9.1)	44.2 (9.4)	45.8 (6.9)	47 (6.9)	44 (7.2)	0.477	0.494	0.970
LAScd (%)	−21.8 (7.7)	−21.7 (7.7)	−23 (7.8)	−24.8 (9.6)	−20.4 (3.7)	0.599	0.301	0.510
LASct (%)	−22.6 (8.4)	−22.4 (8.5)	−23.7 (8.3)	−25.1 (8.7)	−21.7 (8.1)	0.635	0.501	0.845

Value are mean ± SD or *n* (%).

*P*_1_, *P* value between CTRCD vs. no-CTRCD; *P*_2_, *P* value between mild CTRCD vs. moderate-CTRCD; *P*_3_, *P* value between no-CTRCD vs. moderate-CTRCD.

ACE-i, angiotensin-converting enzyme inhibitor; ARB, angiotensin receptor blocker; BMI, body mass index; LAScd, left atrial conduit strain; LASct, left atrial contractile strain; LASr, left atrial resevoir strain; LVEF, left ventricular ejection fraction; LV-GLS, left ventricular global longitudinal strain; RV-FWLS, right ventricular free-wall longitudinal strain; RV-GLS, right ventricular global longitudinal strain.

The bold values mean significant different.

According to the HFA-ICOS risk categories, 52 (47.3%) patients were classified as moderate-risk, and moderate CTRCD occurred only in patients with moderate risk at baseline.

### Left and right heart deformation evolution during trastuzumab chemotherapy

**Figure 2:**
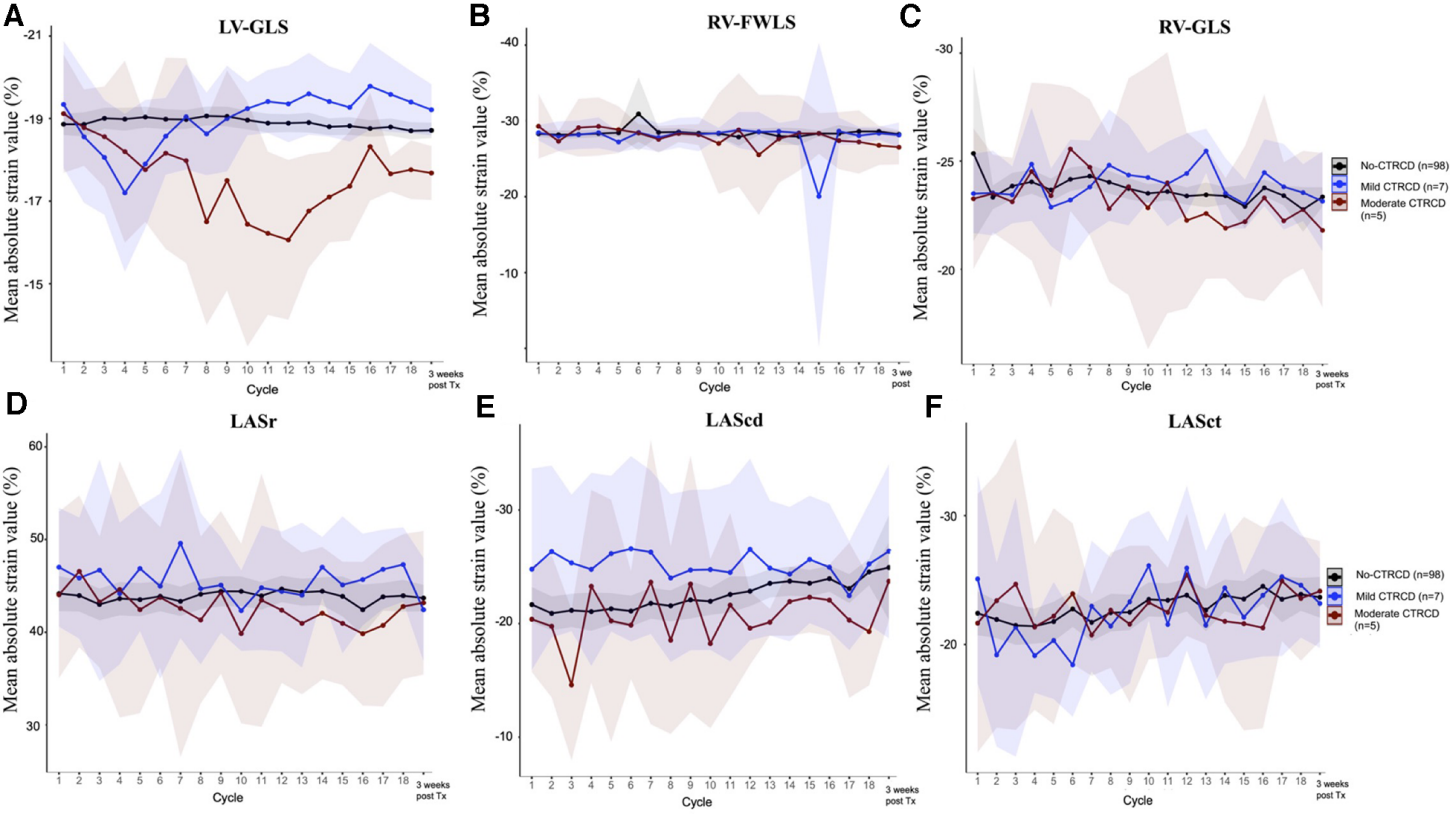
Left heart and right heart strain evolution during trastuzumab chemotherapy LV-GLS decreased significantly in moderate CTRCD (panel **A**), however, other longitudinal strain indices of the right ventricular (RV-FWLS , panel **B** and RV-GLS, panel **C**) and left atrium (LASr, panel **D**; LAScd, panel **E** and LASct, panel **F**) were not affected by trastuzumab cardiotoxicity.

In the CTRCD group, when significant LV-GLS reduction always preceded LVEF reduction, the left atrial longitudinal strain (LASr, LAScd and LASct) and right ventricular longitudinal strain (RV-FWLS and RV-GLS) values did not differ between the CTRCD and no-CTRCD groups or between the mild and moderate CTRCD groups (Figure 2).

**Figure 3:**
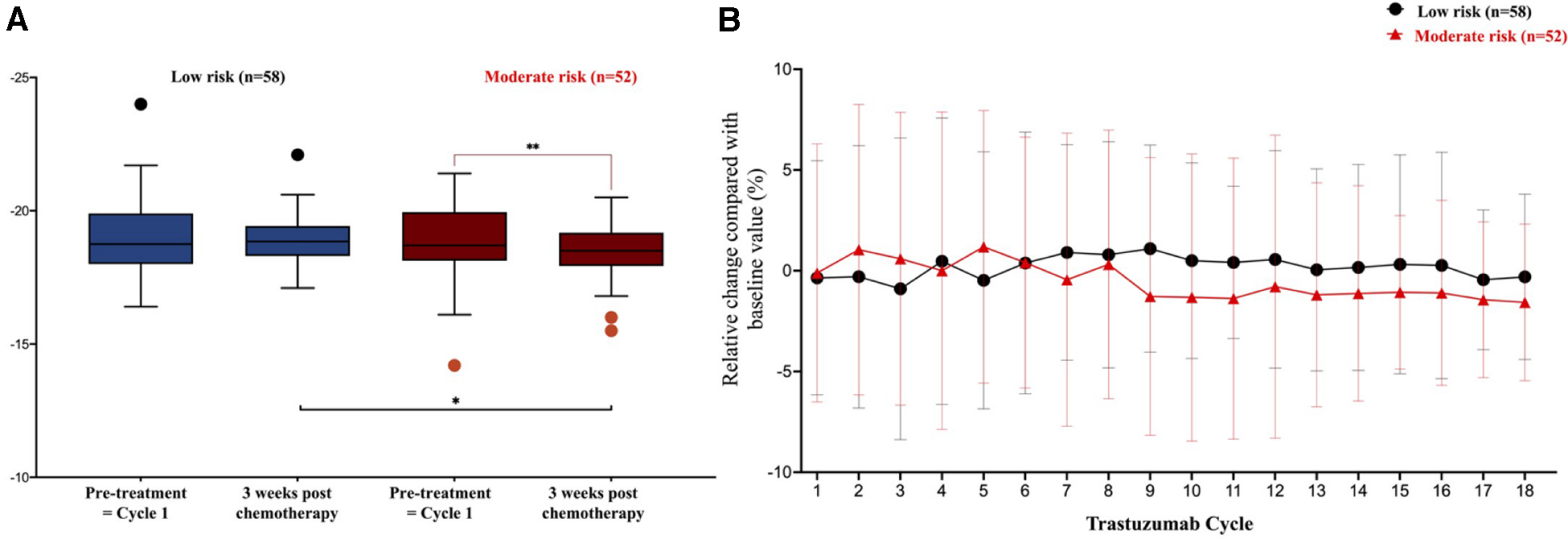
LV-GLS evolution in low risk and moderate risk during trastuzumab chemotherapy. (**A**) In comparison to the low risk, mean LV-GLS in the moderate risk patients were lower at the end of treatment, −18.5 ± 1% vs. −18.9 ± 0.9%, *P *= 0.032. (**B**) Relative changes of LV-GLS between low and moderate risk patients at subsequent cycles of trastuzumab, *P < 0.05. **P < 0.01.

According to the baseline HFA-ICOS risk, moderate-risk patients had a persistent negative relative change in LV-GLS during subsequent trastuzumab cycles, in contrast to the stable trend observed in low-risk patients. Although mean LV-GLS values were similar between the low-risk and moderate-risk patients at baseline, after completing trastuzumab chemotherapy, mean LV-GLS in moderate-risk patients was significantly lower (−18.5 ± 1% vs. −18.9 ± 0.9%; *P* = 0.032) (Figure 3).

At the end of treatment, although LVEF fully improved in all patients with mild and moderate CTRCD, the LV-GLS did not return to the baseline value. In moderate CTRCD patients, the mean LV-GLS at 3 weeks after trastuzumab completion was lower (−17.7 ± 0.5% vs. −19.1 ± 1.2% at baseline; *P* = 0.043). At the time of moderate CTRCD occurrence, the mean LV-GLS and relative reduction of LV-GLS were −15.4 ± 0.7% and 19.0 ± 3.6%, respectively. The lowest LVEF in moderate CTRCD patients was 47.4 ± 1.1%.

## Discussion

When left and right heart myocardial mechanics were concurrently monitored using echocardiography, the most important finding of this prospective study was that CTRCD developed in 10.9% of anthracycline-naïve breast cancer patients undergoing trastuzumab treatment, and half of these CTRCD were mild. However, low- and moderate-risk patients showed different responses to trastuzumab exposure, with more severe CTRCD occurring later and only in non-low-risk patients. In contrast to LV-GLS, right ventricle and left atrial deformation imaging were not sensitive enough to detect the cardiotoxicity induced by trastuzumab when medical therapy for heart failure was implemented in a timely manner.

Although the first signal of CTRCD due to trastuzumab has been recognised for more than 20 years, most of this adverse event was described in the conjunctive chemotherapy protocol with anthracycline (2). In addition, the incidence of trastuzumab-related cardiac dysfunction is inconsistent between clinical trials and observational studies when stringent criteria for patient enrolment and strict protocols of cardiac function monitoring lowered the risk of cardiotoxicity. In trials with non-anthracycline adjuvant (BCIRG-006) (3) or neoadjuvant (TRYPHAENA) (14) regimens, trastuzumab was associated with 0.4% and 1.3% symptomatic heart failure, respectively, but this number could be increased to 14%–27% based on retrospective data that included older patients with cardiovascular diseases treated with trastuzumab (15, 16). Acknowledging the cumulative effect of cardiovascular risk factors on the expression of cardiotoxicity, baseline risk assessment using the HFA-ICOS score was recommended before initiating cancer therapy to individualise cardiac function. However, in contrast to anthracycline, a single algorithm was suggested irrespective of the baseline risk category owing to the lack of validated data in low- or high-risk patients. Although more frequent echocardiography surveillance is performed, a higher likelihood of CTRCD is detected (17), the cost associated with overscreening in the low-risk category is of great concern. Battisti et al. (18) demonstrated that the HFA-ICOS score had a good correlation with increasing rate of cardiac dysfunction, in which the rate of LVEF decline below 50% was 4.5% in low-risk patients and 6.4% in moderate-risk patients; however, most of the patients in this study (65%) were treated concurrently with anthracycline, and cardiotoxicity was defined by LVEF reduction only. In this study, we showed that CTRCD in the low and moderate HFA-ICOS risk categories had different features. The relative decrease in LV-GLS in moderate-risk patients was higher than that in low-risk patients throughout the 18 cycles of trastuzumab treatment. Consequently, at the end of chemotherapy, the mean LV-GLS of moderate-risk patients was significantly lower. In addition, while the incidence of CTRCD was 12.1% and 9.6% in low-risk and moderate- to high-risk patients, respectively, all CTRCD associated with low baseline risk were mild and all CTRCDs occurring in the moderate- to high-risk category had LVEF decline to below 50%. Relative reduction of LV-GLS > 15% in the mild CTRCD in our study did not lead to significant LVEF and LV-GLS reduction after completing trastuzumab treatment, and no heart failure medical treatment was implemented. Since patients treated with trastuzumab are less likely to be associated with guideline-adherent cardiac monitoring compared to other chemotherapy regimens, such as taxane or anthracycline (19), our study suggests that one-size-fits-all echocardiography surveillance every 3 months during the trastuzumab regimen may not be necessary for low-risk patients at baseline. Because all these CTRCD cases developed within the first 6 months, frequent echocardiography can only be considered during this period.

Findings from our study were consistent with data from Battisti (18), Keramida (9), Negishi (20) or Banke (21) in which CTRCD was detected by LV-GLS and/or LVEF reduction in a median time from 5 to 9 months after initiating trastuzumab. While breast cancer patients with a moderate baseline risk were more likely to develop more severe CTRCD, our study suggests that frequent echocardiography surveillance is crucial during the first 9 months of trastuzumab treatment at baseline. In CTRCD of moderate severity, all patients in this study had full recovery at the end of chemotherapy, defined by reversible LVEF with an insignificant reduction of LV-GLS in comparison with the baseline value when medical treatment for heart failure was promptly initiated and trastuzumab chemotherapy was maintained. Although data from previous studies showed that LVEF decline following trastuzumab administration can be persistent (3, 22), this finding from our study confirms the recent ESC guidelines' recommendation regarding safe continuation of anti-HER2 under cardiovascular monitoring if CTRCD can be detected and managed in a timely manner, at least in a moderate setting (5).

To the best of our knowledge, this is the first prospective study to evaluate the role of left atrial and right ventricular deformations in anthracycline-naïve cancer patients undergoing trastuzumab treatment concurrently with LV-GLS. Given the fact that interventricular septum is a major part of left ventricular, RV-GLS evolution is more prone to affected by left ventricular deformation than RV-FWLS (13). Mazzuti et al. showed that RV-GLS decreased during the trastuzumab regimen; however, this was not associated with the CTRCD events (8). After initiating trastuzumab, deterioration of the both right ventricular longitudinal strain parameters (RV-GLS and RV-FWLS) occurred several months following the significant decline of LV-GLS; however, no specific heart failure medical treatment was implemented in the study of Keramida et al. (9) These findings support the result of our study in that when CTRCD was detected and treated early by a significant relative reduction of LV-GLS and mildly reduced LVEF, the chance to recognize any deterioration of isolated right ventricular deformation (RV-FWLS) due to trastuzumab cardiotoxicity is extremely low. Although RV-GLS evolution in moderate CTRCD patients had the similar trend of LV-GLS, mean RV-GLS values at the end of chemotherapy were not different between moderate and no-/mild CTRCD.

For left atrial strain analysis, Park et al. demonstrated that LASr at baseline had a better predictive value for CTRCD than LVGLS after initiating trastuzumab; however, most patients had previously been exposed to anthracycline (10). Similar to right ventricular longitudinal strain, our study showed that no atrial deformation indices (LASr, LAScd or CASct) were sensitive enough to detect trastuzumab-related cardiac dysfunction.

## Study limitations

This study has some limitations. First, no patient was classified into the high-risk HFA-ICOS category. Therefore, our findings may not represent the behaviour of the left and right heart deformations in this group. Second, in the echocardiography protocol, although we assessed LVEF using a well-known semi-automatic software to improve the reproducibility of this measurement, 3D LVEF is the preferred method in recent guidelines owing to its superior accuracy in detecting LV dysfunction. Finally, the incidence of CTRCD was low in our study, which limited the multivariate analysis and the power of Chi-squared test in comparing baseline characteristics among the study groups. Other studies with a considerable proportion of high-risk patients are needed to validate the HFA-ICOS risk category and to identify independent risk factors for early CTRCD development during trastuzumab treatment.

## Conclusions

CTRCD is prevalent in anthracycline-naïve breast cancer patients undergoing trastuzumab chemotherapy, even in non-high-risk categories. However, moderate CTRCD, defined as a reduction in LVEF of <50%, is not encountered frequently. The one-size-fits-all echocardiography surveillance suggested by the recent guidelines is not necessary for low-risk patients.

## Data Availability

The raw data supporting the conclusions of this article will be made available by the authors, without undue reservation.
